# Community-acquired pneumonia: a comparison of clinical treatment failure in patients treated with either penicillin or cefuroxime

**DOI:** 10.1186/1757-7241-20-S2-P10

**Published:** 2012-04-16

**Authors:** Josefin Ekloef, Thomas A Schmidt

**Affiliations:** 1 Department of Emergency Medicine, Holbaek Hospital, Denmark

## Background

National and local guidelines in the Emergency department (ED) at Holbaek hospital recommend penicillin as first-line treatment of community-acquired pneumonia (CAP). Nevertheless, the use of cefuroxime seem to be substantial when admitting patients with CAP.

The aim of this study was to document the use of penicillin and cefuroxime as initial treatment of CAP, and to compare clinical treatment failure (CTF) in the two groups.

## Methods

Data was collected by finding medical records of patients discharged from Holbaek hospital with a principal diagnosis of CAP in the period January-March 2010. A total number of 50 patients were assessed. Patients < 18 years (n=0), with chronic obstructive pulmonary disease (COPD) (n=17), in immune-suppressive treatment (n=4), and receiving other antibiotic as mono therapy (n=2) or as combination therapy (n=5) were excluded.

CTF was defined as change of therapy due to unresponsiveness. Kaplan-Meier plot was used to estimate time until CTF (e.g. end-point) and probability of CTF between the groups. Time to CTF was defined as days from initial therapy to change. Patients dying (n=2) or being discharged without CTF (n=14) were censored at death/discharge.

## Results

All patients were treated with antibiotics intravenously. Treatment was initiated in the ED in 77% of the cases. Fifty-five percent of all patients were treated with cefuroxime. Forty percent of the patients treated with penicillin experienced CTF compared to 17% in the group treated with cefuroxime (p=0.347). Patients were followed for 9 days. At 5 days, a survival rate without CTF was estimated to 0,75 for cefuroxime and 0.54 for penicillin. There was no significant difference between the treatment groups (logrank test: p=0.227). No further failures were seen.

## Conclusion

Cefuroxime was the most common choice of treatment for CAP. Thus, treatment of CAP at Holbaek hospital does not adhere to national or local guidelines. However, numerically the results may indicate a higher rate of CTF in patients treated with penicillin compared to cefuroxime. This need not to be a mere play of chance. A prospective study with a higher number of observations is called for.

